# Rule-Guided Executive Control of Response Inhibition: Functional Topography of the Inferior Frontal Cortex

**DOI:** 10.1371/journal.pone.0020840

**Published:** 2011-06-06

**Authors:** Weidong Cai, Hoi-Chung Leung

**Affiliations:** 1 Department of Psychology, State University of New York at Stony Brook, Stony Brook, New York, United States of America; 2 Department of Psychology, University of California San Diego, La Jolla, California, United States of America; University of Regensburg, Germany

## Abstract

**Background:**

The human inferior frontal cortex (IFC) is a large heterogeneous structure with distinct cytoarchitectonic subdivisions and fiber connections. It has been found involved in a wide range of executive control processes from target detection, rule retrieval to response control. Since these processes are often being studied separately, the functional organization of executive control processes within the IFC remains unclear.

**Methodology/Principal Findings:**

We conducted an fMRI study to examine the activities of the subdivisions of IFC during the presentation of a task cue (rule retrieval) and during the performance of a stop-signal task (requiring response generation and inhibition) in comparison to a not-stop task (requiring response generation but not inhibition). We utilized a mixed event-related and block design to separate brain activity in correspondence to transient control processes from rule-related and sustained control processes. We found differentiation in control processes within the IFC. Our findings reveal that the bilateral ventral-posterior IFC/anterior insula are more active on both successful and unsuccessful stop trials relative to not-stop trials, suggesting their potential role in the early stage of stopping such as triggering the stop process. Direct countermanding seems to be outside of the IFC. In contrast, the dorsal-posterior IFC/inferior frontal junction (IFJ) showed transient activity in correspondence to the infrequent presentation of the stop signal in both tasks and the left anterior IFC showed differential activity in response to the task cues. The IFC subdivisions also exhibited similar but distinct patterns of functional connectivity during response control.

**Conclusions/Significance:**

Our findings suggest that executive control processes are distributed across the IFC and that the different subdivisions of IFC may support different control operations through parallel cortico-cortical and cortico-striatal circuits.

## Introduction

The inferior frontal cortex (IFC) has been associated with a variety of cognitive or executive control processes from target detection, rule retrieval to response control [1], [2], [3], [4], [5], [6], [7]. Among all, the IFC's role in inhibition of inappropriate behavioral responses has received particular emphasis. Previous work on non-human primates demonstrated that damage to the inferior prefrontal convexity could lead to disinhibition of perseverative behaviors [8]. Studies of human adults with frontal lobe damage [9] and transcranial magnetic stimulation (TMS) of the healthy brain [10] have shown that the right inferior frontal gyrus (IFG), in particular the pars opercularis, is critical to the performance of the stop-signal task (SST), which is a cognitive control task requiring the inhibition of prepotent motor responses. In agreement with these findings, neuroimaging studies have shown activations in the posterior IFC during the SST [1], [11], [12], [13], [14], [15], [16], [17].

From the anatomical point of view, the IFC is a large heterogeneous structure consisting of multiple subdivisions with different cytoarchitectonic features and fiber connections in nonhuman primates [18] and humans [19]. Accordingly, the subdivisions are expected to differ in their computational role during executive control of behavior [20]. The comparison of various executive processes has been examined using across-experiment meta analytic approaches [21], [22], [23]. Results from these meta-analyses suggest functional segregation within the IFC by showing that the left anterior IFG is involved in semantic memory retrieval while the right posterior IFG/insula is involved in executive control including response inhibition. Whether or not the IFC has differential functions or a unimodel role in executive control is still in debate [4], [6], [24].

The SST is becoming a popular task for investigating the neural correlates of behavioral inhibition. During the SST, participants make frequent speedy responses to the presentation of the go signal but occasionally withhold their response upon the presentation of the stop signal. Successful inhibition of prepotent responses relies on not only stop-related processes but also other cognitive processes such as infrequent stimulus processing and rule retrieval. Infrequent stimulus processing is accompanied with detecting the stop signal (i.e., the target stimulus) because typically the stop signal is presented in less than a third of the trials during the SST. Recent findings by Chikazoe and colleagues [25] suggested that the right inferior frontal junction (IFJ) is more involved in infrequent stimulus processing rather than response inhibition. Rule retrieval is generally required in sensorimotor tasks; it is the process for retrieving and semantically processing the task-defined stimulus-response associations or action set upon the presentation of the task cue. Some evidence suggests that the left anterior IFC is particularly involved in retrieving and representing of task rules [2], [26].

The stopping process itself involves at least stop-process triggering and successful stopping (i.e., countermanding). We assume the stop process is triggered on all stop trials in order to cancel the initiated motor response, though it may not necessarily lead to successful stopping. Successful stopping would depend on winning the competition between the stop and go processes, as proposed by the race model [27]. While the posterior IFG has been associated with response inhibition, it is unclear whether it is involved in triggering the stop process or countermanding per se [1], [12]. Here, we attempted to directly delineate the functions of the IFC in stopping prepotent responses and differentiate stopping from infrequent stimulus processing and rule retrieval.

**Figure 1:**
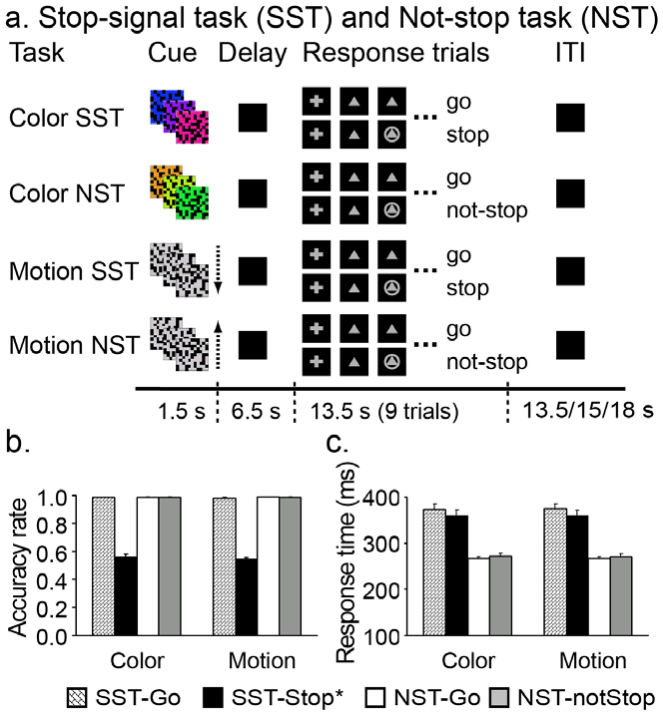
Behavioral tasks and behavioral data. a, A schematic diagram of the task conditions. The present experiment included two visuomotor tasks - a stop signal task (SST) and a not-stop task (NST). Each task was cued by a color and a motion stimulus, forming a total of 4 conditions (color-SST, color-NST, motion-SST and motion-NST). There were three task epochs: cue, delay and a block of response trials. A visual cue (color or motion) was presented at the beginning to indicate the current rule for the response epoch (SST or NST). After a 6.5-sec delay (black screen), a warning signal was presented for 1 sec (not shown) followed by a block of 9 response trials. For both tasks, a go signal was presented on every trial that was occasionally followed by a stop signal (circle) at variable delays (see Methods). Subjects were told to try their best to withhold their response in the presence of the stop signal for the SST but to ignore it (and make their response) for the NST. The inter-task interval varied between 13.5, 15 and 18 sec. b, Average response accuracies for go (SST-Go) and stop (SST-Stop) trials in the SST and go (NST-Go) and not-stop (NST-NotStop) trials in the NST by cue type. c. Average response times across trial types. *SST-Stop refers to all stop trials in b and unsuccessful stop trials in c.

In the present study, we examined the activation of IFG subdivisions in correspondence to the various cognitive processes involved in the SST and analyzed the functional organization of executive control functions in the IFC. To differentiate the multiple cognitive processes in the SST, we incorporated the SST and a parallel visuomotor task (we called it the not-stop task [NST]) (see Figure 1a). The presentation of a task cue and a block of task trials were separated in time. This design allowed us to examine neural activity in correspondence to the cue (rule retrieval) separately from neural activity during the task performance (infrequent target detection and stopping). We separated transient activity from sustained activity during the task block using a mixed general linear model. Sustained activity would be considered representing task-set related functions such as sustained attention and/or implementation of rule and strategy. Transient activity in correspondence to response control was differentiated by comparing trial types. As our interest focused on transient activity, we distinguished between infrequent target detection (comparing stop and not-stop trials relative to go trials) and stop-related steps such as triggering the stop process (comparing unsuccessful stop with not-stop) and successfully stopping (comparing successful with unsuccessful stop trials). We hypothesize that the posterior IFG is involved in stop process, the right dorsal posterior IFG/IFJ in target detection and the left anterior IFG in rule retrieval. Our results revealed that these executive control processes are distributed across the subdivisions of IFG.

## Methods

### Ethics statement

The study protocol was approved by local institutional review board at State University of New York at Stony Brook. All subjects gave written consent.

### Subjects

Twenty-six healthy young adults (age range: 18 – 39 yrs, 11 females, all right handed) were recruited from the Stony Brook University campus and the psychology subject pool, none reported a history of neurological or psychiatric disorders or drug abuse. All subjects had normal or corrected to normal vision. One data set was excluded from the group analysis because the individual's stop accuracy was 3 standard deviations away from the mean and another two were excluded because of image artifacts. Twenty-three subjects were included in the final analysis.

### Behavioral tasks: the stop signal task and not-stop task

This experiment was designed to differentiate brain activity related to response inhibition from activity related to rule retrieval and infrequent target detection under the same experimental setting. The behavioral task was comprised of 2 types of visual cues (color and motion) and 2 types of visuomotor tasks (stop-signal task [SST] and not-stop task [NST]), resulting in 4 cue-task associations (color-SST, color-NST, motion-SST, and motion-NST) (Figure 1a). The SST and NST tasks were visually identical, with the same go and stop signals presented in random sequences but the corresponding response to the stop signal was cue/rule dependent. The color and motion visual cues were used for differentiating regions involved in sensory processing from those involved in cognitive processing (e.g., task rule retrieval), since these two types of visual stimuli are known to elicit responses in anatomically and functionally separable parts of the visual association cortex. Similar color and motion cues were used in previous studies of response inhibition in non-human primates [28].

We used a mixed event-related and block design for our study. Figure 1a shows the timeline of the three task epochs, cue, delay and response. A visual cue was presented at the beginning of each task period indicating the current task for the response block (i.e., SST or NST). After a 6.5-sec delay (black screen), a warning signal was presented for 1 sec followed by a response block of 9 trials. A go signal (500 ms) was presented on each trial. On some trials (about 30%), a stop signal (300 ms) was presented shortly after the go signal. Trial durations varied between 1, 1.5 or 2 sec. Depending on the initial visual cue, subjects would perform either the SST (rule 1: respond to the go signal and withhold the response upon the presentation of the stop signal) or the NST (rule 2: respond to the go signal and ignore the stop signal). In each response block, there were 2, 3 or 4 stop signal trials. The total number of stop signal trials and go trials was equal for the SST and the NST. The trial order in the block is pseudorandomized and counterbalanced across blocks. After the response block, there was a variable resting period (13.5, 15 or 18 sec) before the next visual cue. In order to achieve a stop accuracy of about 50% for the SST, the stop signal delay (SSD), the interval between the go and stop signals, was dynamically adjusted starting from 150 ms. Depending on whether one failed or succeeded on a stop trial, the SSD would be decreased or increased respectively by 50 ms for the next stop trial. The lower and upper limit of the SSD was set at 0 and 600 ms, respectively. On go trials, a correct response required a button press to be registered within 700 ms after the onset of the go signal, whereas on stop trials, a successful stop required no button presses to be registered within 1000 ms after the onset of the go signal. Similar variations in the SSD were used in the NST, with the SSD randomly varied in steps of 50ms ranging from 0 to 400 ms. The various task parameters were counterbalanced across runs within a subject and across subjects. Each experiment included 6 runs. Each run was comprised of 12 task periods, 3 per cue-task association.

### Visual stimuli, Experiment Procedure and Apparatus

The color cues were matrices of black and color squares. The hue of the color cues changed from blue to purple in one and from green to orange in another, with the same range of hue differences. The motion cues were matrices of either upward or downward moving black/grey squares. A fixed set of cue-task associations was randomly assigned to each subject. The associations were counterbalanced across subjects. In both tasks, the go signal was a black triangle and the stop/not-stop signal was the same triangle encircled in a black circle. All stimuli were presented in the center against a light grey background. Each subject was trained a day or two before the scanning session. We first determined an individual's reaction time to the appearance of a visual stimulus (triangle) at random intervals of time for about 2 minutes. Afterwards, he/she was trained to perform the SST and NST tasks for about 40 minutes, first in separate runs and then mixed together as in the real experiment. The training order of the two tasks was counterbalanced across subjects. Subjects were expected to achieve above 95% accuracy on go trials and around 50% accuracy on stop trials in the SST and above 95% accuracy on all trials in the NST. A speedy response to the go signal was emphasized during training and throughout the experiment. On the day of the experiment, each subject was given one run outside of the magnet before performing 6 runs during scanning.

Subjects were given the same instruction “stop when the stop signal is shown in the SST and ignore the stop signal in the NST” from the beginning. Subjects were also required to explain the task to the experimenter before the fMRI session and almost all subjects repeated the task instruction as given in the practice. During the fMRI session, the task instruction was presented on the screen and repeated explicitly by the experimenter before each block.

Visual stimuli were rear-projected onto a screen positioned at the back of the magnet bore opening. Subjects viewed the visual stimuli through a mirror mounted on the head coil. E-prime was used for visual presentation and response data collection (version 2.0.1.109; Psychology Software Tools, Pittsburgh, PA). A response box interfaced with a personal computer's parallel port was used for collecting the manual responses.

### SSRTs estimation

Following the Race Model [27], we estimated the stop signal reaction time (SSRT) based on the inhibition function (which is the probability of responding on stop trials as a function of SSD) and the distribution of RT for go trials. Since the stop accuracy of most subjects was not exactly at 50%, SSRT was estimated using the integration method. The following equation was used: SSRT = T-SSD, where T was the point when the integration of go RT equals to the proportion of unsuccessful stop trials. To minimize biases caused by the extreme SSDs [29], the final SSRT of each individual was obtained by averaging the SSRT estimates from 2 to 3 central SSDs with the most observations (about 25–30 trials per SSD). (There were about 5 to 8 SSDs per individual.)

### Statistical analysis of behavioral data

To determine the effect of sensory cue and task on behavioral performance, we applied two-way ANOVA (cue [color vs. motion] x task [SST vs. NST]) to test for task differences in go accuracy, go reaction time, and stop/not-stop accuracy. Two-way ANOVA (cue [color vs. motion] x trial type [go vs. not-stop trials]) was also conducted to examine differences in RT between the two trial types in the NST.

### Image Acquisition

All scans were conducted on a Philips 3 T Achieva system with an eight-channel SENSE head coil (Cleveland, OH). Head movement was minimized using foam padding and a tape across the forehead. For every subject, we first collected a series of high-resolution structural 3D images (T1-weighted, 3D turbo field echo, 176 sagittal slices, slice thickness = 1 mm, TR/TE = 9.9/4.6 ms, matrix = 256×256, FOV = 25×25 cm) and then a series of T1-weighted inplane structural images, parallel to the anterior-posterior commissural (AC–PC) line (24 axial slices, slice thickness = 5 mm, TR/TE = 300/5.0 ms, Matrix = 256×256, FOV = 22×22 cm). Six series of functional images were acquired along the same AC–PC plane using a standard single shot echo planar pulse sequence (24 axial slices, interleaved, 5-mm thick, TR/TE = 1500/30 ms, Matrix = 64×64, FOV = 22×22 cm, Flip angle = 80o, 309 volumes/session [463.5 sec]).

### Image Preprocessing

Images were first screened for obvious artifacts such as ghosting and motion. Runs with images showing large motion and artifacts were removed from further analysis. One run was removed from one data set because of image artifact and three runs were removed from another data set because of excess motion. Images were processed using Statistical Parametric Mapping version 2 (SPM2, Welcome Department of Imaging Neuroscience, University College London, http://www.fil.ion.ucl.ac.uk/spm/). The first four images of each series of functional scans were discarded to take into account the time required for T1 signal to reach equilibrium. Images were corrected for differences in timing of slice acquisition and head motion. Functional series with images of greater than 3 mm of translational and 1.5o of rotational motion were excluded from data analysis. A mean functional image volume was generated for each individual using the realigned images. The inplane and high-resolution 3D anatomical images were co-registered with the mean functional image and segmented into grey and white matter. The segmented grey matter of the inplane image was then normalized to the Montreal Neurological Institute (MNI) grey matter template, using a 12-parameter affine registration followed by a series of nonlinear transformations. The normalization parameters were then applied to all the realigned functional images. Finally, all functional images were spatially smoothed with a Gaussian kernel of 8 mm at full-width at half maximum and were high-pass filtered with a cutoff at 1/128 Hz.

### Image Data Modeling

Blood oxygenation level-dependent (BOLD) signal in correspondence to the task epochs and events were estimated using the general linear model (GLM) [30]. For each individual dataset, a model was constructed including the following regressors for each of the four task conditions (color/motion SST/NST): cue, first half of the delay, second half of the delay, response block and the various trial types (i.e., go, stop, not-stop) in the SST and NST. Other factors (e.g., warning) and potential confounds (e.g. error in go trials) were included in the model as effects of no interest.

More specifically, the cue regressor was the onset times of the cue presentation for each task. The delay epoch was modeled by two regressors coding the first and second half of the delay in accordance with methods used in studies of delayed recognition [31]. The delay epoch separated the cue and response epochs by 6.5 seconds. Since the cue and the first half of delay were only 1.5 sec apart, the second delay vector was used to examine delay-related brain activity. The response epoch was modeled as a mixture of block and event regressors to derive sustained task effects and transient trial effects, respectively [32]. The block regressors of the SST and NST were constructed using the onset times and duration of the response blocks (with the block onset and offset times as separate vectors in the model independent of the task conditions). The following trial types were modeled as events: go (SST-Go), successful stop (SST-succStop) and unsuccessful stop (SST-unsuccStop) in the SST and go (NST-Go) and not-stop (NST-notStop) in the NST. (Since initial tests showed little or no sensory-related effects in the response epoch, the color and motion conditions of each task were combined in the analysis of response block and trials.) We chose to use this mixed block and event model for eliminating or minimizing potential confounds in between-block comparisons (e.g. SST-unsuccStop vs. NST-notStop), such as differences in sustained attention, overall task difficulty and task strategy. For validation purposes, we conducted additional analysis to derive the transient effects in correspondence to the trial types in the SST/NST without the block regressors (i.e., using an event-only GLM). Since the whole brain contrast maps from the event-only model and the mixed block and event model revealed similar activation patterns, we only presented the maps from the mixed model. This is not surprising because the SST and NST shared the common resting periods and they were presented in pseudorandom sequences within each run and were counterbalanced across runs.

All vectors were convolved with a canonical hemodynamic response function (HRF) and entered as regressors in the GLM. To eliminate artifacts caused by task-related motion, six motion parameters were entered as covariates. This procedure was demonstrated to increase the signal-to-noise ratio and improve task effects estimated using the GLM [33].

### Voxel-wise individual and group image analysis

Estimated parameters (beta values) of each task epoch and event were derived for each individual using the GLM described above (i.e., first-level analysis). T tests were applied at the group level for comparing and contrasting task blocks and events (i.e., second-level analysis). Unless otherwise stated, a threshold of p<0.05 (FDR corrected) was used to generate contrast maps.

We focused on differentiating the transient effects in correspondence to stopping and other cognitive processes (infrequent target detection and rule retrieval) using a series of contrasts. We first identified the overall transient effects of response control in the SST by comparing all the stop trials (SST-allStop) with the go trials. Transient effects of stopping were examined by comparing SST-succStop and SST-Go. The stopping effect was further confirmed by directly comparing the SST-allStop versus SST-Go contrast and the NST-notStop versus NST-Go contrast. Because of double subtraction, the results were masked by the SST-allStop versus SST-Go contrast (the mask was generated at p<0.05, uncorrected; cluster size > = 9). We then systematically examined activity related to successful stopping using the SST-succStop and SST-unsuccStop contrast, as responses were withheld on the successful stop trials but not on the unsuccessful stop trials. Activity related to the triggering of stop processes was examined by comparing SST-unsuccStop with NST-notStop because the two types of trials shared similar sensory inputs and motor outputs and the main difference between them was that subjects presumably put in a greater effort to stop the prepotent responses during SST-unsuccStop but not so during NST-notStop. Activity related to infrequent target detection was examined using the conjunction of two contrasts (NST-notStop versus NST-Go and SST-allStop versus SST-Go) as both NST-notStop and SST-allStop were equally infrequent. The conjunction analysis identified the significant activation over both contrasts [34], [35]. For conjunction analysis, the same threshold was applied for both contrasts at p<0.001, uncorrected, which gives a joint probability of 0.00001.

### Regions of interest (ROI) analysis

To further test our hypotheses regarding the particular involvement of posterior IFG in the stop process and the right dorsal IFG in infrequent stimulus processing, we defined 3 ROIs in the IFG that have been associated with different cognitive processes in previous studies. The bilateral ventral-posterior IFG (left: x = −42, y = 12, z = −6; right: x = 42, y = 18, z = −6) were defined from our previous study in which they were activated in stopping both hand and eye movements [15]. It is important to point out that the ventral-posterior ROI may contain parts of both posterior IFG and insula and the activation of these two areas are hard to separate at this particular location. The right dorsal-posterior IFG/IFJ (x = 48, y = 4, z = 38) was selected from a study in which it was associated with infrequent stimulus processing rather than response inhibition using a Go/NoGo task [25]. The center of this ROI is at the junction of inferior frontal sulcus and precentral sulcus, commonly called IFJ in some studies. All ROIs were spheres of 6-mm radius centered at the coordinates listed above. The beta values of each task epoch and event were derived for each ROI for each subject using Marsbar (http://marsbar.sourceforge.net/). The beta values from different trial conditions were compared using paired t-test and corrected for multiple comparisons.

### Functional connectivity analysis

Psychophysiological interaction (PPI) analysis was applied to examine the interactions between IFG subdivisions and other brain areas during the SST block in comparison to the NST block [36], [37]. The PPI analysis has been validated as a robust method for detecting functional connectivity between brain regions in block designs [38]. Hence, results from our PPI analysis can only be interpreted as the overall differences in functional connectivity between the two tasks. Coordinates of the seed regions were determined individually, guided by the group contrast of the SST and NST blocks (see Tables S3 and S4). For each individual, we used the SST-NST contrast at an uncorrected threshold of p<0.05 to find the suprathreshold voxel nearest to the peak coordinate of the group. Volumes of interest (VOI) were spheres with 6-mm radius, which was half of the distance between the centers of the two closest clusters in the group contrast map. The physiological component (Y series) was extracted for each VOI, corrected for variance associated with parameter of no interest and deconvolved with the HRF. The psychological component (P series) was generated by convolving the contrast of the SST versus NST with the HRF. The psychological interaction component (PPI series) was derived by reconvolving the multiplication of the physiological component and psychological component with the HRF. The PPI, Y and P series were used as predictors in the regression analysis. The PPI group analysis were conducted at a threshold of p<0.05, FDR corrected. For the purpose of visualization, we presented PPI group maps of all VOIs at a combination of activation threshold (p<0.001, uncorrected) and cluster filter (9 contiguous voxels), which has been suggested to be sufficient to reduce the probability of type 1 error [39].

## Results

### Behavioral results

Figure 1b shows the accuracy and reaction time results. No significant main effect was found for the visual-cue (color/motion) factor in either the SST or the NST (all F_1,22_<1.1, p>0.3, Figure 1b). The main effect of task was not significant for go accuracy (F_1,22_<1.1, p>0.3), but was significant for go RT, with responses slower to SST-Go (374±55 ms) than to NST-Go (mean ± SD: 265±21 ms) (F_1,22_ = 116, p<0.001, Figure 1c), suggesting additional processing (e.g., preparing to stop) occurred on go trials in the SST. The average RT for NST-notStop (272±25 ms) was slightly longer than that for NST-Go; the slight difference (about 7 ms) suggests that the participants detected the stop signal and followed the not-stop rule in the NST (F_1,22_ = 14, p<0.001). The average SSRT was estimated to be 158±7 ms in the SST, and as expected, the average SSD for SST-succStop trials (166±61 ms) were significantly shorter than that for SST-unsuccStop trials (218±70 ms), t_22_ = 17.73, p<0.001.

### fMRI results

We examined results from the whole-brain, ROI and functional connectivity analyses to determine the extent to which the different subdivisions of IFC (and other brain regions) contribute to stop-related processes in comparison to other cognitive processes such as infrequent target detection (i.e., stop-signal detection) and rule retrieval. Brain activity during the various trial types in the SST and NST blocks was used to characterize the cognitive control processes in the IFC. Since sustained control processes are expected to be engaged during task performance [40], we used the mixed block/event model to separate transient brain activities from sustained activities.

### Whole brain analysis: IFG and stopping

**Figure 2:**
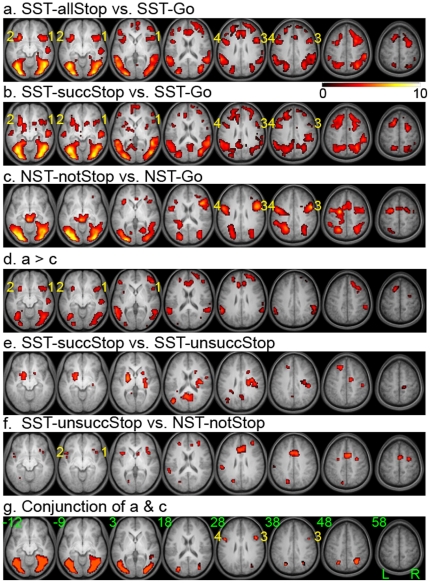
Group contrast maps showing transient activations during response control. a. Widespread activations shown by contrasting all stop trials and go trials of the SST. b. Activations revealed by contrasting the successful stop trials and go trials of the SST. c. Activations shown by contrasting the not-stop trials and go trials of the NST. Note that less parts of IFC showed suprathreshold activation. d. Activations revealed by directly comparing a and c. e. Activations revealed by contrasting the successful and unsuccessful stop trials of the SST. f. Activations revealed by contrasting the unsuccessful stop trials of the SST and the not-stop trials of the NST. These two types of trials were similar in both frequency and motor output. g. Conjunction maps showing activations that are suprathreshold in both contrasts, SST-allStop versus SST-Go and NST-notStop versus NST-Go. Within the IFC, suprathreshold activation was only observed in the dorsal posterior IFG. All the contrast maps were thresholded at p<0.05 (FDR corrected) and overlaid on anatomical images averaged across the group. The numbers in the bottom row indicate the z level of the transverse slices in mm. Activation labels: 1, right ventral-posterior IFG; 2, left ventral-posterior IFG; 3, right dorsal-posterior IFG/IFJ; 4, left dorsal-posterior IFG/IFJ.

As a starting point, we examined the “stop-go” contrast (SST-allStop vs. SST-Go; Figure 2a) since it is commonly used in many previous response inhibition studies. As expected, the activation results are similar to previous findings using the same contrast [15], [17] (see Table S1 for the complete list of activation clusters). The stop-go contrast revealed activations in various parts of the IFC that are clearly differentiable (local maxima > = 12 mm apart), including the bilateral ventral-posterior IFG (extending to anterior insula), bilateral dorsal-posterior IFG/IFJ, bilateral anterior IFG, and right dorsal-anterior IFG (extending to MFG). These IFG areas remained suprathreshold even after the SST-unsuccStop trials were removed, as shown in the SST-succStop versus SST-Go contrast (Figure 2b). In comparison, the ventral-posterior IFG showed little or no responses in the NST-notStop versus NST-Go contrast while the dorsal-posterior IFG/IFJ showed similar responses as in the stop-go contrast (Figure 2c). Direct comparison between the SST-allStop versus SST-Go contrast and the NST-notStop versus NST-Go contrast confirmed that the ventral-posterior IFG is indeed more involved in stopping whereas the dorsal-posterior IFG is involved in both stopping and not-stopping (p<0.05, FDR corrected, Figure 2d). It is worth to mention that these two contrasts are matched in stop-signal frequency.

Since the stop-go contrast alone includes not only stopping processes but also other control processes, we applied several contrasts to differentiate the role of IFG in stopping. To test whether a particular subdivision of the IFG contributes to successful stopping or active countermanding [1], [41], we examined the SST-succStop versus SST-unsuccStop contrast. Most parts of the IFC including the ventral-posterior IFG were about equally active during both SST-succStop and SST-unsuccStop. Instead, the bilateral ventral striatum, left dorsal prefrontal cortex, right posterior insula and posterior cingulate cortex (PCC) are more active during the successful stop trials in comparison to the unsuccessful stop trials in the SST (Figure 2e). We did not find any suprathreshold activations in the opposite contrast (SST-unsuccStop > SST-succStop) at the same threshold. In sum, these data suggest that rather than direct countermanding, the IFG areas play some other roles in stopping.

### Whole brain analysis: IFG and stop triggering

If the ventral-posterior IFG was not involved in direct countermanding, what role does it play in stopping? We applied the contrast of SST-unsuccStop versus NST-notStop to examine whether the ventral-posterior and dorsal-anterior IFG areas are related to the triggering or initiation of the stop processes in response to the stop signal. These two types of trials shared the same visual input (stop signals were presented on both trials) and motor output (motor responses were made on both trials), but differed in that the subjects endeavored to inhibit their motor responses (i.e. stop process was nonetheless triggered) on the SST-unsuccStop trials but not so on the NST-notStop trials. Indeed, the bilateral ventral-posterior IFG/insula and the right dorsal-anterior IFG (extending to MFG) were more active during SST-unsuccStop compared to NST-notStop (Figure 2f). These activations in the IFG cannot be simply error related, since the same IFG areas were equally active during successful stop (see above and Figure 2b). In comparison, the activations found in the ACC (extending to SMA) in the SST-unsuccStop versus NST-notStop contrast was also found in the SST-unsuccStop versus SST-succStop contrast at a lower threshold. Detail discussions on the role of ACC in error and performance monitoring can be found elsewhere [42]. The opposite contrast, NST-notStop versus SST-unsuccStop, did not show any significant activation in the IFG. In sum, these findings suggest that the ventral-posterior IFG/insula is involved in processes other than direct countermanding such as stop triggering.

### Whole brain analysis: IFG and stop-signal detection

Recent literature suggests that the right IFJ is involved in processing the infrequent stimulus rather than response stopping [25]. We used the contrast of NST-notStop versus NST-Go and the conjunction of two contrasts (NST-notStop versus NST-Go and SST-allStop versus SST-Go) to isolate activity related to infrequent target detection (i.e., the stop signal). Aside from the visual cortical regions, the bilateral dorsal-posterior IFG/IFJ, supplementary motor area (SMA), PPC and premotor cortex were more active during NST-notStop compared to NST-Go (Figure 2c). The activation in the dorsal-posterior IFG/IFJ was extensive. Since the NST-notStop and SST-allStop trials were equally infrequent and their occurrence patterns were closely matched, the conjunction of the NST-notStop versus NST-Go and SST-allStop versus SST-Go contrasts further confined the activations in correspondence to infrequent target detection to the bilateral dorsal-posterior IFG/IFJ (Figure 2g).

### ROI analysis: Activity patterns of the IFG subregions during stopping and not-stopping

**Figure 3:**
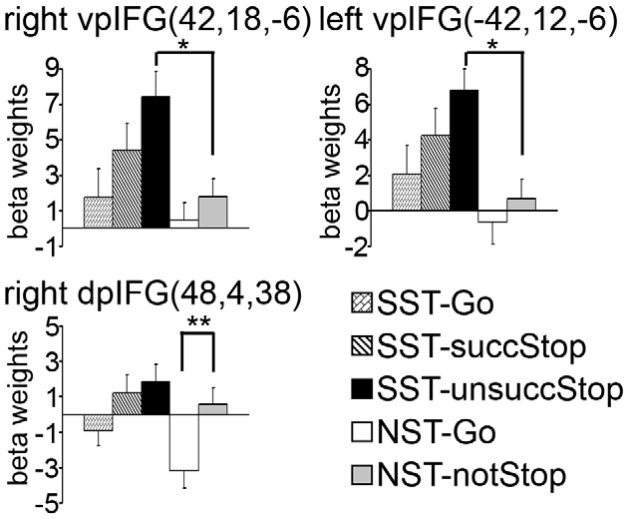
Activation patterns of pre-defined IFG ROIs during response control across trial types. Three ROIs were defined using coordinates reported in previous neuroimaging studies of relevant topics, including right vpIFG, left vpIFG and right dpIFG/IFJ. In each bar chart, the bars from left to right show the average beta weights in correspondence to the various trial types: SST-Go, SST-succStop, SST-unsuccStop, NST-Go, and NST-notStop. Error bars show the standard error. The coordinates (x, y, z) are in mm. *, p<0.05; **, p<0.01, corrected for multiple comparisons. All ROIs showed significantly stronger activity on stop trials (SST-allStop) in comparison to go trials (not marked). Abbreviations: vpIFG: ventral-posterior IFG; dpIFG: dorsal-posterior IFG.

To further test the differential functions of IFG subdivisions, we defined a set of ROIs based on previous studies of response inhibition and evaluated their activation pattern during the SST and NST in the present study (Figure 3). As expected, all ROIs showed significant activity in the comparison of SST-allStop and SST-Go (p's<0.05). More specifically, ROIs in the left and right ventral-posterior IFG (regions previously found related to response inhibition independent of effector by Leung and Cai [15]) showed significant differences in activity between SST-unsuccStop and NST-notStop (p<0.05). The right ventral-posterior IFG ROI further exhibited stronger activity in SST-succStop than SST-Go (p = 0.05) and in SST-unsuccStop than SST-succStop, whereas the same comparisons did not reach significance in the left ventral-posterior IFG after Bonferroni correction for multiple comparisons (all p's>0.1). ROIs in the right dorsal-posterior IFG/IFJ (regions previously found related to infrequent stimulus processing by Chikazoe et al. [25]) showed significant differences in activity in both NST-notStop versus NST-Go and SST-allStop versus SST-Go (p's<0.01). These results were corrected for the number of multiple comparisons.

### Whole-brain analysis: IFG and rule retrieval

Brain activity in response to the task cue presentation was used to determine whether the IFG areas involved in stopping or target detection are also involved in rule retrieval. Regions involved in rule retrieval were expected to show task-cue dependent (SST vs. NST) activity irrespective to the visual features of the cues (color vs. motion). Whole brain analysis revealed only a small suprathreshold activation in the left middle temporal gyrus (MTG), which showed stronger responses to both color and motion cues for the NST in comparisons to those for the SST (p<0.05, FDR corrected). At a lower threshold (p<0.001, uncorrected), the same contrast revealed activations in the left anterior IFG, the frontopolar cortex and the right MTG, but not in the ventral or dorsal posterior or dorsal anterior part of the IFG. See Table S1 for clusters and coordinates. To further examine differential cue-related activity within the IFG, we applied SVC (small volume correction) to the NST-SST contrast at the cue stage using the IFG mask from the AAL atlas (automated anatomical labeling atlas, WFU_PickAtlas by Advanced Neuroscience Imaging Research Lab, Winston-Salem, NC; http://www.fmri.wfubmc.edu/download.htm) and found significant activations only in the left anterior IFG (p<0.05, FDR corrected). (As expected, sensory-dependent activation was found in the left fusiform gyrus (FG) and bilateral posterior MTG for color and motion processing, respectively [p<0.05, FDR corrected]). It is worth mentioning that we did not observe suprathreshold activity during the delay period for either the SST or NST.

### Functional connectivity of the IFG subregions during the SST in comparison to the NST

**Figure 4:**
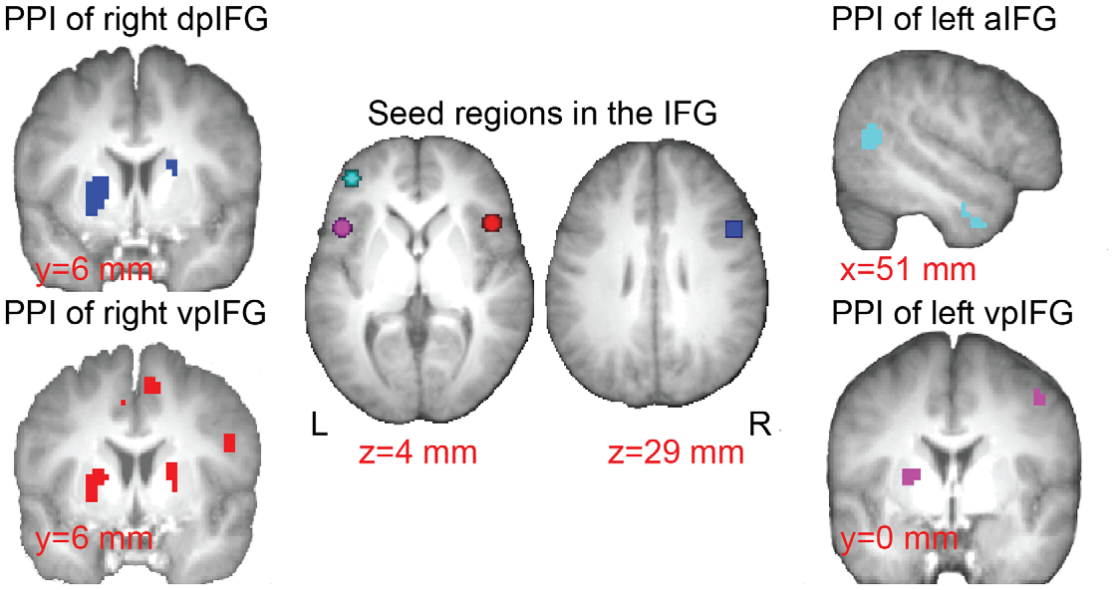
Psychophysiological interactions of IFG subregions during response control. The images in the center illustrate the IFG subregions on the axial slices. The functional connectivity of each IFG region during the response blocks of SST versus NST is shown on the side (neurological orientation: right = right). The right dpIFG (average MNI coordinates: x = 51, y = 14, z = 29) showed stronger interactions with the striatum. The left aIFG (x = −42, y = 46, z = 7) showed stronger interactions with the right middle temporal gyrus. The right vpIFG (x = 47, y = 20, z = 0) showed stronger interactions with striatum, premotor cortex and supplementary motor area. The left vpIFG (x = −47, y = 17, z = 0) showed stronger interactions with striatum and premotor cortex. All PPI maps were presented at threshold of p<0.001, uncorrected. Abbreviations: aIFG: anterior inferior frontal gyrus; vpIFG: ventral-posterior IFG; dpIFG: dorsal-posterior IFG.

We conducted psychophysiological interaction (PPI) analysis to examine the interactions between the IFG regions and other brain areas during the SST in comparison with the NST. Since we used the entire response block for the PPI analysis, these results can only be interpreted as the general differences in functional connectivity during the performance of the two tasks. As shown in Figure 4, the IFG regions showed similar yet different patterns of functional connectivity. Stronger connectivities were found between the right dorsal-posterior IFG/IFJ and the putamen and cerebellum (p<0.05, FDR corrected). At a lower threshold, similar functional connectivity with the putamen and cerebellum was evident for the right and left ventral-posterior IFG/anterior insula (p<0.001, uncorrected). The left anterior IFG showed a stronger coupling with the right MTG during the SST compared to the NST (p<0.05, FDR corrected). See Table S2 for the complete list of clusters.

**Figure 5:**
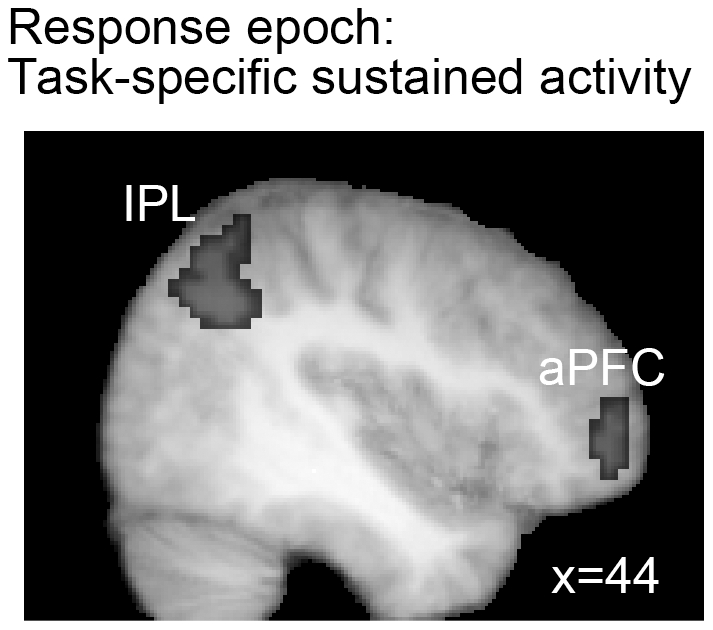
Task-dependent effects during the response epoch. Greater sustained activations during the response epoch of the SST in comparison to the NST are shown on a sagittal slice (p<0.05, FDR corrected). Abbreviations: aPFC: anterior prefrontal cortex; IPL: inferior parietal lobule.

### Whole brain analysis: Task-specific sustained effects during response control

As shown in Figure 5, the right anterior PFC and inferior parietal lobule (IPL) (more specifically, the angular gyrus) showed greater sustained activation during the SST block than during the NST block (p<0.05, FDR corrected). These task-dependent changes in sustained activation during the response epoch are probably related to differences in the level of sustained attention and maintenance and/or implementation of task rule and strategy. Previous research has shown sustained activity in the anterior PFC in association with sustained cognitive control [40] and active maintenance of task rules [26]. Detailed discussions on the role of the right anterior PFC and IPL in task-set maintenance can be found elsewhere [43].

## Discussion

Our results demonstrate that the executive control functions such as response inhibition, infrequent target detection and rule retrieval are distributed across the IFC. The functional subdivisions of IFC seem to have similar but also distinct patterns of functional connectivity during response control. These findings suggest that the IFC subdivisions may support different cognitive operations involved in executive control through parallel cortico-cortical and cortico-striatal circuits.

### Inhibiting prepotent motor responses: preparing, triggering and stopping

Previous studies have emphasized that the posterior part of the right IFC (particularly pars opercularis) plays a critical role in response inhibition [9], [10]. In the present study, we distinguished the stop-related processes from rule retrieval and infrequent target detection and the functions of IFG subdivisions in stopping prepotent motor responses. Our findings suggest that the ventral part of posterior IFG in both hemispheres (and the right dorsal anterior IFG) are more involved in triggering the stop process rather than stopping per se. This suggestion is based on the finding of greater activation in the ventral posterior IFG during SST-unsuccStop in comparison to NST-notStop. These two types of trials were well matched in both sensory and motor domains; however, the subjects presumably attempted to inhibit the planned response on the unsuccessful stop trials in the SST but not so on the not-stop trials in the NST. In other words, the stop process, albeit lost in the competition to the go process, was nonetheless initiated or triggered on the unsuccessful stop trials (see race model [27], which suggests that successful stop is determined by a faster stop process in comparison to the go process). In corroboration, it has been recently demonstrated that the ventral posterior IFG is even activated under conditions when subjects were unaware of the presentation of the no-go signals but nevertheless slowed down their responses as if stopping was triggered [44].

One may argue that the activations revealed by the SST-unsuccStop and NST-notStop contrast might have been associated with error-related processes such as error detection and feedback processing [45]. Our ROI analysis showed that the right ventral-posterior IFG was slightly more activated during the SST-unsuccStop trials than during the SST-succStop trials. However, since the SSDs were much longer for the SST-unsuccStop trials relative to the SST-succStop trials, factors other than error processing such as lengthened stop-signal processing, stop preparation [46] and difficulty to stop could also contribute to the observed differences in activity between the two types of stop trials in the SST. While it is difficult to rule out error-related effects, it is less likely to be the case because the bilateral ventral-posterior IFG were also significantly more active on successful stop trials in comparison to go trials. Furthermore, previous studies focused on studying error-related processes did not find the posterior-ventral IFG to be particularly involved in error detection in the stop-signal task [17], [41], [47]. Instead, many have shown that the ventral-posterior IFG is not sensitive to the successfulness of stopping behavior [1], [12], [41], though some found that the ventral-posterior IFG is more involved in successful than unsuccessful inhibition [17]. Notably, the go RT and SSRT were much longer (about 800 ms and 300 ms, respectively) in the latter study [17] than those (about 400 ms and 190 ms, respectively) in the former study [1], indicating between-group differences in preparation or performance strategies during the SST. Another study found that the right IFG was more activated during successful than unsuccessful stopping in the whole-brain analysis but the difference was insignificant in the ROI analysis [7]; the cautionary note from this latter study is that it is potentially too conservative to use just the contrast between successful and unsuccessful stopping for determining the neural substrates involved in response inhibition because the two types of stop trials share most of sensory and cognitive processes required by the SST.

Another consideration is whether the right IFC is more specifically involved in stopping unwanted responses or more generally related to the attentional demand, which has been a topic of concern in recent studies [4], [6], [48]. To dissociate stopping from attentional effects, these studies have utilized both an infrequent signal relevant to the stopping behavior and an infrequent signal not directly relevant to the stopping behavior in their designs similar to the current study. Some suggested that the involvement of the right IFC during stopping is associated with the detection of salient target stimuli (i.e., the stop signal) instead of response inhibition [4], [6]. Findings from the present study and a previous study [7], however, are in favor of the hypothesis that the ventral-posterior IFG is more involved in response stopping while the dorsal posterior IFG is more involved in target detection (see below). There are two potential explanations for the different patterns of right IFC activation observed across studies. First, even though all experiments described above were designed to include a not-stopping behavioral requirement, there was no guarantee that inhibition was not engaged at all at the neural level. In fact, the RT for the not-stop trials (called “continue trial”) was 40 ms slower than that for the go trials in the Sharp et al. study [6]. The slower response to the stimulus for the infrequent not-stop trials may reflect the engagement of response inhibition or other executive processes. In contrast, the RT difference between the NST-notStop trials and the NST-Go trials in the current study (7 ms) as well as the RT difference between the Stop-irrelevant stop trials and the Stop-irrelevant go trials in the Boehler et al. study [7] (3 ms) were minimum among this group of studies. Second, in both the current study and the Boehler et al. study [7], the infrequent stimulus on the not-stop trials was not directly associated with large behavioral change like the stop trials or the “counting” and “respond” conditions in the Hampshire et al. study. We are not excluding the possibility that the right ventral-posterior IFC is to certain degree modulated by salient and significant behavioral change. Rather, the right IFC seems more sensitive to action updating demands [49]. Perhaps the ventral-posterior IFG is at the intersection between attention and response control through interacting with the visual association regions to enhance attention to the stop signal [50] and with the striatum to trigger response countermanding [1], respectively. Indeed, the activation in the ventral-posterior IFG in the present study is in the same vicinity as the area that we previously found independent of the visual feature of the stop signal [12] and response modality [15].

Taking the various lines of findings together, we suggest that the ventral-posterior IFG may not be directly responsible for stopping or blocking prepotent responses, but for processes at the early stage of stopping, such as preparing and triggering the stop process. In a recent TMS study, Verbruggen et al. [49] used theta burst stimulation to temporally disrupt the function of right dorsal and ventral IFG while subjects performed a stop-signal task (similar to the SST in our study), a stop-ignore task (similar to the NST in our study) and a dual-signal task in which an additional response is required upon the presentation of a dual signal. They found that the right ventral IFG is critical for updating action plans (whereas the right dorsal IFG is critical for visual detection of changes in the environment), though neither was related to the actual stopping process itself. Then, which region(s) is(are) actually responsible for suppressing the motor cortex and withholding the motor response? Though our experiment does not provide a definitive answer to this question, the striatum and dorsomesial prefrontal cortex showed greater activation during successful stopping than during unsuccessful stopping in the present study and were functionally connected with the IFG regions during the SST. It is likely that the actual stopping process involves the subcortical regions and preSMA as indicated by the recent literature [1], [11], [51].

It is also important to point out that the ventral-posterior IFG cluster extends to the anterior insula in the group contrast maps as well as in previous neuroimaging studies using the stop-signal task [1], [11], [12], [15]. The anterior insula has been associated with performance monitoring and error awareness [52]. Although it would be interesting to functionally dissociate the ventral-posterior IFG and anterior insula in response inhibition, the current study cannot provide a clear distinction of their functions.

### Target detection during response control

In contrast to the ventral-posterior IFG, the dorsal part of the posterior IFG or IFJ in both hemispheres showed activation patterns in correspondence to infrequent target detection. These findings are comparable with a recent fMRI study using a variant of a go/no-go task [25], in which the investigators found increased activity in the right IFJ during both the infrequent go and no-go trials in comparison to the frequent go trials. Our findings further demonstrate that the bilateral dorsal-posterior IFG/IFJ is involved in processing the infrequent task-relevant stop signals in the SST as well as the infrequent task-irrelevant stop signal in the NST. Other investigators have reported activations in this part of the IFG in response to both task-relevant and task-irrelevant infrequent stimuli [53], [54], [55]. However, it should be noted that the “task-irrelevant” stimulus might not be totally irrelevant if ignoring a salient stimulus was part of the task rule, especially in conjunction with recent experiences when the stimulus was relevant. Furthermore, although the activation of dorsal-posterior IFG/IFJ was about equal during the stop and not-stop trials, a stronger correlation was found between the dorsal-posterior IFG/IFJ and putamen during the performance of the SST in comparison to the NST. This suggests that the dorsal-posterior IFG/IFJ is not passively responding to the stop signal but actively involved in using the stop signal to guide response stopping through interacting with the motor regions. This region has been also associated with cognitive control studies of task switching and set shifting [56]. Although the exact role of the dorsal-posterior IFG or IFJ in cognitive control remains to be determined, the current findings together with existing evidence suggest that this region may play an important role in stimulus-driven reorientation of attention and thus contribute to both top-down and bottom-up processes during executive control of behavior [47].

### Rule retrieval

In comparison to the ventral-posterior IFG and dorsal-posterior IFG, we found that the left anterior IFG exhibited activation patterns in correspondence to rule retrieval and is functionally connected with right MTG during the SST. Previous non-human primate studies have demonstrated that the IFC is involved in learning and retrieving stimulus-response associations [28], [57], [58], [59]. Human brain imaging studies have also shown that the left IFG is involved in retrieving and representing task rules [2], [26]. Consistent with these previous findings, we found that the left anterior IFG (mainly the ventral-anterior pars triangularis) is more active in correspondence to the color/motion cues representing the NST compared to the SST. Although the NST may seem easier than the SST at the sensorimotor control level, the NST cues may involve additional semantic representation for the associated condition-action rule provided to the subjects (i.e., “ignore the stop signal and respond like the go trials” as oppose to “don't respond in the presence of the stop signal”). Perhaps this region is modulated by the level of cue-rule relations [60], [61]. While the greater function connectivity between left anterior IFG and MTG during the response block of the SST appears to be contradictory to the greater activation of left anterior IFG during the cue epoch of NST, it could also be explained by the greater demand of rule retrieval and maintenance during performing the SST in comparison to the NST. Indeed, the MTG is often associated with retrieving the meaning of tool words, action words and traffic signs from long-term memory [62], [63], [64]. This functional connectivity suggests that aside from stimulus-rule association, the left anterior IFG is also involved in retrieving the behavioral meaning of the stop signal during task performance, probably through interacting with the right MTG.

In sum, our findings demonstrate that executive control functions are distributed across the IFC, with the ventral-posterior IFG/anterior insula for the process at the early stage of stopping (e.g. triggering the stop process), the dorsal-posterior IFG/IFJ for infrequent target detection and the left anterior IFG for rule retrieval. The actual countermanding seems to be outside of the IFC. The subdivisions of IFC may play their various roles in executive control through interacting with the striatum, cerebellum and frontal motor regions. These results reveal the functional topography of the IFC as well as the possible parallel cortico-cortical and cortico-striatal circuits during rule-guided executive control of motor behavior.

## Supporting Information

Table S1 List of clusters and coordinates from the group contrasts shown in Figure 2 of the main text.
(DOC)

Table S2 List of clusters and coordinates from the PPI analysis.
(DOC)

Table S3 List of PPI seed coordinates for individuals.
(DOC)

Table S4 List of clusters and coordinates from the contrast of SST-NST response blocks (using block GLM).
(DOC)

## References

[1] Aron AR, Poldrack RA (2006). Cortical and subcortical contributions to Stop signal response inhibition: role of the subthalamic nucleus.. J Neurosci.

[2] Bunge S, Kahn I, Wallis J, Miller E (2003). Neural circuits subserving the retrieval and maintenance of abstract rules.. J Neurophysiol.

[3] Garavan H, Ross TJ, Stein EA (1999). Right hemispheric dominance of inhibitory control: an event-related functional MRI study.. Proc Natl Acad Sci USA.

[4] Hampshire A, Chamberlain SR, Monti MM, Duncan J, Owen AM (2010). The role of the right inferior frontal gyrus: inhibition and attentional control.. Neuroimage.

[5] Konishi S, Nakajima K, Uchida I, Kikyo H, Kameyama M (1999). Common inhibitory mechanism in human inferior prefrontal cortex revealed by event-related functional MRI.. Brain 122 (Pt.

[6] Sharp DJ, Bonnelle V, De Boissezon X, Beckmann CF, James SG (2010). Distinct frontal systems for response inhibition, attentional capture, and error processing.. Proc Natl Acad Sci U S A.

[7] Boehler CN, Appelbaum LG, Krebs RM, Hopf JM, Woldorff MG (2010). Pinning down response inhibition in the brain--conjunction analyses of the Stop-signal task.. Neuroimage.

[8] Iversen SD, Mishkin M (1970). Perseverative interference in monkeys following selective lesions of the inferior prefrontal convexity.. Experimental brain research Experimentelle Hirnforschung Expérimentation cérébrale.

[9] Aron AR, Fletcher PC, Bullmore ET, Sahakian BJ, Robbins TW (2003). Stop-signal inhibition disrupted by damage to right inferior frontal gyrus in humans.. Nat Neurosci.

[10] Chambers CD, Bellgrove MA, Stokes MG, Henderson TR, Garavan H (2006). Executive "brake failure" following deactivation of human frontal lobe.. Journal of cognitive neuroscience.

[11] Aron AR, Behrens TE, Smith S, Frank MJ, Poldrack RA (2007). Triangulating a cognitive control network using diffusion-weighted magnetic resonance imaging (MRI) and functional MRI.. J Neurosci.

[12] Cai W, Leung H-C (2009). Cortical activity during manual response inhibition guided by color and orientation cues.. Brain Res.

[13] Congdon E, Mumford JA, Cohen JR, Galvan A, Aron AR (2010). Engagement of large-scale networks is related to individual differences in inhibitory control.. Neuroimage.

[14] Coxon JP, Stinear CM, Byblow WD (2009). Stop and go: the neural basis of selective movement prevention.. Journal of cognitive neuroscience.

[15] Leung H-C, Cai W (2007). Common and differential ventrolateral prefrontal activity during inhibition of hand and eye movements.. J Neurosci.

[16] Rubia K, Russell T, Overmeyer S, Brammer MJ, Bullmore ET (2001). Mapping motor inhibition: conjunctive brain activations across different versions of go/no-go and stop tasks.. Neuroimage.

[17] Rubia K, Smith AB, Brammer MJ, Taylor E (2003). Right inferior prefrontal cortex mediates response inhibition while mesial prefrontal cortex is responsible for error detection.. Neuroimage.

[18] Petrides M, Pandya DN (2002). Comparative cytoarchitectonic analysis of the human and the macaque ventrolateral prefrontal cortex and corticocortical connection patterns in the monkey.. Eur J Neurosci.

[19] Croxson PL, Johansen-Berg H, Behrens TEJ, Robson MD, Pinsk MA (2005). Quantitative investigation of connections of the prefrontal cortex in the human and macaque using probabilistic diffusion tractography.. J Neurosci.

[20] Passingham RE, Stephan KE, Kötter R (2002). The anatomical basis of functional localization in the cortex.. Nat Rev Neurosci.

[21] Cabeza R, Nyberg L (2000). Neural bases of learning and memory: functional neuroimaging evidence.. Curr Opin Neurol.

[22] Simmonds DJ, Pekar JJ, Mostofsky SH (2008). Meta-analysis of Go/No-go tasks demonstrating that fMRI activation associated with response inhibition is task-dependent.. Neuropsychologia.

[23] Wager TD, Sylvester C-YC, Lacey SC, Nee DE, Franklin M (2005). Common and unique components of response inhibition revealed by fMRI.. Neuroimage.

[24] Aron AR (2009). Introducing a special issue on stopping action and cognition.. Neurosci Biobehav Rev.

[25] Chikazoe J, Jimura K, Asari T, Yamashita K-i, Morimoto H (2009). Functional dissociation in right inferior frontal cortex during performance of go/no-go task.. Cereb Cortex.

[26] Sakai K, Passingham RE (2006). Prefrontal set activity predicts rule-specific neural processing during subsequent cognitive performance.. J Neurosci.

[27] Logan GD, Cowan WB (1984). On the Ability to Inhibit Thought and Action: A Theory of an Act of Control.. Psychol Rev.

[28] Sakagami M, Ki T, Lauwereyns J, Koizumi M, Kobayashi S (2001). A code for behavioral inhibition on the basis of color, but not motion, in ventrolateral prefrontal cortex of macaque monkey.. J Neurosci.

[29] Band GPH, van der Molen MW, Logan GD (2003). Horse-race model simulations of the stop-signal procedure.. Acta psychologica.

[30] Friston KJ (1995). Statistical Parametric Maps in Functional Imaging: A General Linear Approach.. Human brain mapping.

[31] Zarahn E, Aguirre G, D'Esposito M (1997). A trial-based experimental design for fMRI.. Neuroimage.

[32] Visscher K, Miezin F, Kelly J (2003). Mixed blocked/event-related designs separate transient and sustained activity in fMRI.. Neuroimage.

[33] Johnstone T, Ores Walsh KS, Greischar LL, Alexander AL, Fox AS (2006). Motion correction and the use of motion covariates in multiple-subject fMRI analysis.. Human brain mapping.

[34] Friston KJ, Penny WD, Glaser DE (2005). Conjunction revisited.. Neuroimage.

[35] Nichols T, Brett M, Andersson J, Wager T, Poline J-B (2005). Valid conjunction inference with the minimum statistic.. Neuroimage.

[36] Friston KJ, Buechel C, Fink GR, Morris J, Rolls E (1997). Psychophysiological and modulatory interactions in neuroimaging.. Neuroimage.

[37] Gitelman DR, Penny WD, Ashburner J, Friston KJ (2003). Modeling regional and psychophysiologic interactions in fMRI: the importance of hemodynamic deconvolution.. Neuroimage.

[38] Kim J, Horwitz B (2008). Investigating the neural basis for fMRI-based functional connectivity in a blocked design: application to interregional correlations and psycho-physiological interactions.. Magn Reson Imaging.

[39] Poline JB, Worsley KJ, Evans AC, Friston KJ (1997). Combining spatial extent and peak intensity to test for activations in functional imaging.. Neuroimage.

[40] Braver TS, Reynolds JR, Donaldson DI (2003). Neural mechanisms of transient and sustained cognitive control during task switching.. Neuron.

[41] Li C-sR, Huang C, Constable RT, Sinha R (2006). Imaging response inhibition in a stop-signal task: neural correlates independent of signal monitoring and post-response processing.. J Neurosci.

[42] Ullsperger M, von Cramon DY (2001). Subprocesses of performance monitoring: a dissociation of error processing and response competition revealed by event-related fMRI and ERPs.. Neuroimage.

[43] Dosenbach NU, Visscher KM, Palmer ED, Miezin FM, Wenger KK (2006). A core system for the implementation of task sets.. Neuron.

[44] van Gaal S, Ridderinkhof KR, Scholte HS, Lamme VAF (2010). Unconscious activation of the prefrontal no-go network.. J Neurosci.

[45] Hirose S, Chikazoe J, Jimura K, Yamashita K, Miyashita Y (2009). Sub-centimeter scale functional organization in human inferior frontal gyrus.. Neuroimage.

[46] Chikazoe J, Jimura K, Hirose S, Yamashita K-i, Miyashita Y (2009). Preparation to Inhibit a Response Complements Response Inhibition during Performance of a Stop-Signal Task.. J Neurosci.

[47] Chevrier AD, Noseworthy MD, Schachar R (2007). Dissociation of response inhibition and performance monitoring in the stop signal task using event-related fMRI.. Hum Brain Mapp.

[48] Aron AR, Durston S, Eagle DM, Logan GD, Stinear CM (2007). Converging evidence for a fronto-basal-ganglia network for inhibitory control of action and cognition.. J Neurosci.

[49] Verbruggen F, Aron AR, Stevens MA, Chambers CD (2010). Theta burst stimulation dissociates attention and action updating in human inferior frontal cortex.. Proc Natl Acad Sci USA.

[50] Corbetta M, Shulman GL (2002). Control of goal-directed and stimulus-driven attention in the brain.. Nat Rev Neurosci.

[51] Li C-sR, Yan P, Sinha R, Lee T-W (2008). Subcortical processes of motor response inhibition during a stop signal task.. Neuroimage.

[52] Ullsperger M, Harsay HA, Wessel JR, Ridderinkhof KR (2010). Conscious perception of errors and its relation to the anterior insula.. Brain Struct Funct.

[53] Huettel SA, McCarthy G (2004). What is odd in the oddball task? Prefrontal cortex is activated by dynamic changes in response strategy.. Neuropsychologia.

[54] Kirino E, Belger A, Goldman-Rakic P, McCarthy G (2000). Prefrontal activation evoked by infrequent target and novel stimuli in a visual target detection task: an event-related functional magnetic resonance imaging study.. J Neurosci.

[55] Linden DE, Prvulovic D, Formisano E, Völlinger M, Zanella FE (1999). The functional neuroanatomy of target detection: an fMRI study of visual and auditory oddball tasks.. Cereb Cortex.

[56] Brass M, Derrfuss J, Forstmann B, von Cramon DY (2005). The role of the inferior frontal junction area in cognitive control.. Trends Cogn Sci.

[57] Bussey TJ, Wise SP, Murray EA (2001). The role of ventral and orbital prefrontal cortex in conditional visuomotor learning and strategy use in rhesus monkeys (Macaca mulatta).. Behav Neurosci.

[58] Rushworth MFS, Buckley MJ, Gough PM, Alexander IH, Kyriazis D (2005). Attentional selection and action selection in the ventral and orbital prefrontal cortex.. J Neurosci.

[59] Wang M, Zhang H, Li BM (2000). Deficit in conditional visuomotor learning by local infusion of bicuculline into the ventral prefrontal cortex in monkeys.. Eur J Neurosci.

[60] Christoff K, Gabrieli JD (2000). The frontopolar cortex and human cognition: Evidence for a rostrocaudal hierarchical organization within the human prefrontal cortex.. Psychobiology.

[61] Simons JS, Owen AM, Fletcher PC, Burgess PW (2005). Anterior prefrontal cortex and the recollection of contextual information.. Neuropsychologia.

[62] Chao LL, Haxby JV, Martin A (1999). Attribute-based neural substrates in temporal cortex for perceiving and knowing about objects.. Nat Neurosci.

[63] Donohue SE, Wendelken C, Crone EA, Bunge SA (2005). Retrieving rules for behavior from long-term memory.. Neuroimage.

[64] Martin A, Haxby JV, Lalonde FM, Wiggs CL, Ungerleider LG (1995). Discrete cortical regions associated with knowledge of color and knowledge of action.. Science.

