# Supplementary material for: Rule-Guided Executive Control of Response Inhibition: Functional Topography of the Inferior Frontal Cortex
Source: PLoS One. 2011 Jun 6;6(6):e20840. doi: 10.1371/journal.pone.0020840 (PMC3108978; doi:10.1371/journal.pone.0020840)
Supplement: Table S1 — List of clusters and coordinates from the group contrasts shown in Figure 2 of the main text. (DOC) [file pone.0020840.s001.doc]

Supplementary Table 1 List of clusters and coordinates from the group contrasts shown in Figure 2 of the main text.

| regions | X | Y | Z | cluster size | Z |
| --- | --- | --- | --- | --- | --- |
|  |  |  |  |  |  |
| *Cue Epoch* |  |  |  |  |  |
| NST > SST |  |  |  |  |  |
| middle temporal gyrus | -60 | -51 | -3 | 4 | 4.68 |
| anterior inferior frontal gyrus* | -48 | 33 | 12 | 48 | 3.5 |
|  |  |  |  |  |  |
| Color > Motion |  |  |  |  |  |
| fusiform gyrus | -33 | -66 | -18 | 66 | 4.68 |
|  |  |  |  |  |  |
| Motion > Color |  |  |  |  |  |
| middle temporal gyrus | 48 | -63 | -3 | 99 | 5.7 |
| middle temporal gyrus | -48 | 69 | 0 | 79 | 4.89 |
|  |  |  |  |  |  |
| *Response Epoch* |  |  |  |  |  |
| SST-allStop > SST-Go |  |  |  |  |  |
| inferior temporal gyrus | -27 | -90 | -12 | 2779 | 5.83 |
| middle temporal gyrus | -39 | -63 | -9 |  | 5.42 |
| middle occipital gyrus | -33 | -90 | 9 |  | 5.39 |
| inferior temporal gyrus | 45 | -66 | -15 | 4868 | 5.76 |
| inferior temporal gyrus | 45 | -54 | -18 |  | 5.54 |
| inferior occipital gyrus | 33 | -84 | -9 |  | 5.39 |
| insula | -30 | 18 | -15 | 1104 | 4.8 |
| anterior inferior frontal gyrus | -51 | 39 | 12 |  | 4.41 |
| superior temporal gyrus | -45 | 12 | -30 |  | 3.91 |
| cingulate gyrus | -12 | 27 | 27 | 475 | 4.08 |
| cingulate gyrus | 9 | 33 | 24 |  | 3.88 |
| cingulate gyrus | 6 | 45 | 3 |  | 3.09 |
| superior frontal sulcus | -24 | 39 | 36 | 179 | 3.9 |
| middle frontal gyrus | -27 | 42 | 21 |  | 3.44 |
| middle frontal gyrus | -27 | 51 | 30 |  | 3.27 |
| amygdala | 18 | -3 | -21 | 25 | 2.98 |
| fusiform gyrus | -24 | -15 | -33 | 12 | 2.96 |
| superior colliculus/midbrain | -3 | -24 | -3 | 27 | 2.87 |
| amygdala | -18 | -9 | -21 | 21 | 2.8 |
| cuneus | 9 | -63 | 6 | 15 | 2.62 |
| parieto-occipital fissure | 18 | -57 | 9 |  | 2.36 |
| Inferior frontal gyrus sub-clusters |  |  |  |  |  |
| anterior inferior frontal gyrus | -51 | 39 | 12 |  | 4.41 |
| anterior inferior frontal gyrus | 48 | 45 | 3 |  | 3.11 |
| ventral-posterior inferior frontal gyrus / anterior insula (2) | -42 | 12 | -9 |  | 3.2 |
| ventral-posterior inferior frontal gyrus / anterior insula (1) | 36 | 12 | -6 |  | 3.78 |
| ventral-posterior inferior frontal gyrus (1) | 51 | 15 | 3 |  | 3.48 |
| dorsal-posterior inferior frontal gyrus / inferior frontal junction (4) | -48 | 9 | 27 |  | 3.39 |
| dorsal-posterior inferior frontal gyrus / inferior frontal junction (3) | 48 | 8 | 30 |  | 3.65 |
| dorsal-anterior inferior/middle frontal gyrus | 33 | 42 | 30 |  | 2.91 |
|  |  |  |  |  |  |
| SST-succStop > SST-Go |  |  |  |  |  |
| middle occipital gyrus | -36 | -69 | -9 | 10320 | 5.89 |
| middle temporal gyrus | 39 | -72 | 12 |  | 5.73 |
| middle occipital gyrus | -30 | -90 | -12 |  | 5.71 |
| cerebellum | -15 | -60 | -39 | 33 | 3.73 |
| superior frontal sulcus | 24 | -3 | 60 | 731 | 3.71 |
| superior frontal gyrus | 21 | 6 | 55 |  | 3.51 |
| superior frontal gyrus | 24 | 9 | 48 |  | 3.46 |
| middle frontal gyrus | 33 | 21 | 51 |  | 3.4 |
| frontal pole | 33 | 39 | 39 |  | 3.14 |
| frontal pole | 12 | 54 | 36 |  | 2.89 |
| dorsal-posterior inferior frontal gyrus / inferior frontal junction | 48 | 9 | 30 |  | 2.82 |
| superior frontal gyrus | 12 | 33 | 48 |  | 3.59 |
| middle frontal gyrus | 27 | 6 | 51 |  | 3.51 |
| anterior inferior frontal gyrus | -51 | 39 | 12 | 72 | 3.44 |
| anterior inferior frontal gyrus | -48 | 39 | 0 |  | 3.41 |
| ventral-posterior inferior frontal gyrus | 57 | 21 | 6 | 32 | 3.17 |
| anterior inferior frontal gyrus | 51 | 39 | 0 | 32 | 3.04 |
| thalamus | 9 | -6 | 6 | 23 | 2.96 |
| posterior commissure | -3 | -24 | -3 | 17 | 2.64 |
| supplementary motor cortex | 9 | -9 | 51 | 24 | 2.61 |
| cingulate gyrus | 9 | 0 | 42 |  | 2.56 |
|  |  |  |  |  |  |
| NST-notStop > NST-Go |  |  |  |  |  |
| inferior occipital gyrus | -45 | -69 | -6 | 4565 | 5.77 |
| inferior occipital gyrus | -27 | -84 | -12 |  | 5.71 |
| inferior occipital gyrus | -39 | -78 | -12 |  | 5.63 |
| superior frontal gyrus | -21 | -6 | 48 |  | 5.29 |
| precentral gyrus | -51 | 3 | 27 |  | 4.34 |
| precentral gyrus | -42 | -3 | 42 |  | 4.24 |
| precentral gyrus | -48 | -6 | 36 |  | 4.06 |
| frontal eye field | -30 | -3 | 63 |  | 4.01 |
| supplementary motor area | -12 | -3 | 69 |  | 3.37 |
| inferior occipital gyrus | 33 | -84 | -6 | 2293 | 5.73 |
| fusiform gyrus | 42 | -63 | -15 |  | 5.35 |
| dorsal-posterior Inferior frontal gyrus / inferior frontal junction | 48 | 9 | 33 |  | 5.15 |
| fusiform gyrus | 45 | -54 | -18 |  | 4.96 |
| inferior frontal gyrus | 39 | 18 | 18 |  | 4.85 |
| inferior frontal gyrus | 48 | 27 | 12 |  | 4.12 |
| superior frontal gyrus | 21 | -6 | 69 |  | 4.12 |
| superior frontal gyrus | 21 | 3 | 63 |  | 3.95 |
| superior parietal lobule | 30 | -57 | 45 |  | 3.77 |
| brain stem | -6 | -30 | -12 | 314 | 5.05 |
| hippocampus | -21 | -27 | -6 |  | 3.5 |
| anterior insula | -33 | 24 | 6 | 104 | 4.15 |
| cingulate gyrus | 3 | 30 | 3 | 10 | 3.15 |
| superior temporal gyrus | -48 | 6 | -21 | 11 | 2.9 |
| lingual gyrus | -12 | -72 | 6 | 22 | 2.85 |
| middle frontal gyrus | 24 | 42 | 18 | 28 | 2.83 |
| middle frontal gyrus | 30 | 36 | 21 |  | 2.77 |
| superior temporal gyrus | -48 | -42 | 12 | 9 | 2.73 |
|  |  |  |  |  |  |
| SST-allStop - SST-Go > NST-notStop - NST-Go ** |  |  |  |  |  |
| temporal pole | 39 | 6 | -24 | 311 | 4.44 |
| orbital frontal cortex | 27 | 21 | -18 |  | 4.03 |
| temporal pole | 45 | 15 | -15 |  | 3.19 |
| orbital frontal cortex | -30 | 18 | -15 | 101 | 4.29 |
| insula | -33 | 12 | 0 |  | 2.48 |
| ventral-posterior inferior frontal gyrus / anterior insula | -39 | 9 | -6 |  | 2.38 |
| supramarginal gyrus | -60 | -48 | 27 | 1009 | 4.08 |
| middle temporal gyrus | -57 | -45 | 3 |  | 3.85 |
| fusiform gyrus | -36 | -60 | -9 |  | 3.77 |
| anterior inferior frontal gyrus | 48 | 39 | 0 | 353 | 3.97 |
| frontal pole | 21 | 57 | 30 |  | 3.78 |
| frontal pole | 39 | 48 | 6 |  | 3.29 |
| fusiform gyrus | 42 | -66 | -15 | 890 | 3.88 |
| fusiform gyrus | 42 | -54 | -18 |  | 3.66 |
| middle temporal gyrus | 57 | -51 | 6 |  | 3.59 |
| cingulate gyrus | -9 | 27 | 27 | 192 | 3.81 |
| cingulate gyrus | 9 | 36 | 24 |  | 3.53 |
| cingulate gyrus | 0 | 30 | 18 |  | 3.15 |
| frontal pole | -48 | 42 | 12 | 57 | 3.57 |
| superior frontal gyrus | -18 | 18 | 57 | 54 | 3.51 |
| superior frontal gyrus | 18 | 30 | 51 | 88 | 3.46 |
| middle frontal gyrus | 30 | 21 | 48 |  | 3.27 |
| temporal pole | -48 | 12 | -30 | 10 | 3.4 |
| supramarginal gyrus | 63 | -27 | 24 | 20 | 3.09 |
| middle temporal gyrus | 54 | -18 | -12 | 72 | 3.09 |
| frontal pole | -27 | 48 | 27 | 49 | 3.03 |
| superior parietal lobule | -15 | -60 | 54 | 8 | 2.77 |
| middle frontal gyrus | 33 | 6 | 51 | 27 | 2.68 |
| superior frontal gyrus | 27 | -3 | 57 |  | 2.62 |
|  |  |  |  |  |  |
| SST-succStop > SST-unsuccStop |  |  |  |  |  |
| putamen | -21 | -3 | -3 | 382 | 4.91 |
| hippocampus | -27 | -15 | -18 |  | 4.08 |
| unknown | -15 | 6 | -15 |  | 3.97 |
| white matter | 33 | -6 | 24 | 539 | 4.35 |
| insula sulcus | 39 | -15 | 21 |  | 4.24 |
| putamen | 30 | -21 | -3 |  | 4.23 |
| cingulate gyrus | -9 | -54 | 15 | 251 | 4.13 |
| cingulate gyrus | 0 | -60 | 21 |  | 4 |
| white matter | 15 | -45 | 21 |  | 3.11 |
| middle occipital gyurs | -48 | -75 | 18 | 96 | 4.09 |
| precentral gyrus | 12 | -30 | 63 | 44 | 4.03 |
| superior frontal gyrus | 21 | -18 | 66 |  | 3.12 |
| lateral fissure | -33 | -39 | 24 | 34 | 4.01 |
| white matter | -36 | -48 | 9 |  | 3.53 |
| white matter | -24 | -48 | 30 |  | 3.13 |
| supplementary motor cortex | 12 | -9 | 51 | 33 | 3.84 |
| middle temporal gyrus | 51 | -51 | 12 | 18 | 3.65 |
| putamen | 21 | 12 | 0 | 26 | 3.65 |
| superior frontal sulcus | -15 | 30 | 48 | 78 | 3.65 |
| superior frontal sulcus | -24 | 30 | 48 |  | 3.27 |
| white matter | 21 | 15 | 27 | 28 | 3.5 |
| precentral gyrus | 51 | -3 | 15 | 12 | 3.37 |
| unknown | 12 | 9 | -15 | 13 | 3.28 |
| precuneus | -6 | -57 | 42 | 11 | 3.27 |
| lingual gyrus | -12 | -48 | 0 | 10 | 3.23 |
|  |  |  |  |  |  |
| SST-unsuccStop > NST-notStop |  |  |  |  |  |
| superior temporal gyrus | 39 | 9 | -24 | 156 | 4.37 |
| ventral-posterior inferior frontal gyrus / anterior insula | 33 | 27 | 0 |  | 3.67 |
| insula | 33 | 18 | 6 |  | 3.61 |
| intraoccipital sulcus/middle occipital gyrus | -24 | -69 | 24 | 76 | 4.31 |
| intraparietal sulcus | -18 | -69 | 39 |  | 3.73 |
| intraparietal sulcus | -12 | -75 | 51 |  | 3.2 |
| supplementary motor cortex | 0 | 0 | 54 | 459 | 4.2 |
| cingulate gyrus | -6 | 12 | 42 |  | 4.1 |
| cingulate gyrus | 9 | 9 | 48 |  | 4.1 |
| precentral gyrus | 30 | -3 | 51 | 57 | 3.99 |
| superior frontal gyrus | 18 | -3 | 69 |  | 3.19 |
| dorsal-anterior inferior/middle frontal gyrus | 33 | 42 | 24 | 54 | 3.88 |
| supramarginal gyrus | -54 | -30 | 24 | 86 | 3.86 |
| postcentral gyrus | -60 | -24 | 33 |  | 3.47 |
| superior temporal gyrus | -57 | -42 | 15 |  | 3.36 |
| cuneous/lingual gyrus | -21 | -72 | 6 | 13 | 3.75 |
| middle frontal gyrus | -27 | 39 | 21 | 21 | 3.74 |
| precentral gyrus | -24 | -3 | 45 | 12 | 3.64 |
| insula | -39 | 12 | 0 | 66 | 3.62 |
| superior temporal gyrus | -54 | 12 | -6 |  | 3.37 |
| superior temporal gyrus | -48 | 0 | -6 |  | 3.34 |
| fusiform gyrus | -39 | -54 | -21 | 41 | 3.58 |
| fusiform gyrus | -33 | -57 | -15 |  | 3.19 |
| ventral-posterior inferior frontal gyrus / anterior insula | -30 | 27 | -3 | 12 | 3.53 |
| caudate | 9 | 12 | 3 | 12 | 3.38 |
|  |  |  |  |  |  |
| conjunction of SST-allStop - SST-Go and NST-notStop - NST-Go |  |  |  |  |  |
| inferior occipital gyrus | -30 | -78 | -12 | 598 | 5.18 |
| inferior occipital gyrus | -30 | -87 | -15 |  | 5.17 |
| inferior occipital gyrus | -42 | -69 | -6 |  | 5.12 |
| inferior occipital gyrus | 33 | -84 | -9 | 415 | 4.8 |
| inferior occipital gyrus | 42 | -66 | -15 |  | 4.7 |
| inferior occipital gyrus | 45 | -54 | -18 |  | 4.68 |
| superior parietal lobule | -21 | -60 | 45 | 54 | 4.19 |
| dorsal-posterior inferior frontal gyrus / inferior frontal junction | 48 | 9 | 30 | 71 | 3.98 |
| dorsal-posterior inferior frontal gyrus / inferior frontal junction | -51 | 6 | 27 | 10 | 3.3 |

All activation peaks were suprathreshold at p<0.05, FDR corrected, except

* Left anterior IFG showed task-related (NST>SST) activation in the cue epoch at a lower threshold, p<0.001 uncorrected; cluster size >= 9. The activation survived FDR corrected threshold of p<0.05 when applied a small volume correction using a IFG mask derived from the Automated Anatomical Labeling (AAL) atlas.

** Because of the double subtraction, the results were masked by the SST-allStop versus SST-Go contrast (the mask was generated at p<0.05, uncorrected; cluster size >= 9).
