# Supplementary material for: Rule-Guided Executive Control of Response Inhibition: Functional Topography of the Inferior Frontal Cortex
Source: PLoS One. 2011 Jun 6;6(6):e20840. doi: 10.1371/journal.pone.0020840 (PMC3108978; doi:10.1371/journal.pone.0020840)
Supplement: Table S2 — List of clusters and coordinates from the PPI analysis. (DOC) [file pone.0020840.s002.doc]

Supplementary Table 2 List of clusters and coordinates from the PPI analysis

| regions | X | Y | Z | cluster size | Z |
| --- | --- | --- | --- | --- | --- |
|  |  |  |  |  |  |
| *seed: left aIFG* |  |  |  |  |  |
| middle temporal gyrus / superior temporal sulcus | 51 | -57 | 15 | 15 | 4.65 |
| middle temporal gyrus / superior temporal sulcus | 42 | -60 | 12 |  | 4.16 |
|  |  |  |  |  |  |
| seed: right dpIFG |  |  |  |  |  |
| cerebellum | -33 | -60 | -36 | 47 | 4.72 |
| cerebellum | 30 | -57 | -33 | 150 | 4.5 |
| cerebellum | 9 | -54 | -18 |  | 4.07 |
| cerebellum | 18 | -51 | -27 |  | 3.95 |
| putamen | -21 | 6 | 3 | 26 | 4.07 |
| putamen | 21 | 12 | 15 | 9 | 3.66 |
|  |  |  |  |  |  |
| seed: right vpIFG * |  |  |  |  |  |
| cerebellum | 30 | -60 | -33 | 207 | 4.1 |
| cerebellum | 6 | -63 | -18 |  | 4 |
| cerebellum | 12 | -54 | -24 |  | 4 |
| cerebellum | -36 | -57 | -39 | 22 | 3.9 |
| supplementary motor area | 9 | 3 | 54 | 130 | 3.9 |
| supplementary motor area | -3 | -12 | 66 |  | 3.8 |
| supplementary motor area | -3 | 0 | 48 |  | 3.7 |
| precentral gyrus | -42 | -12 | 54 | 39 | 3.7 |
| postcentral gyrus | -33 | -27 | 51 |  | 3.3 |
| putamen | -24 | 3 | 0 | 67 | 3.6 |
| putamen | 21 | 6 | 6 | 46 | 3.6 |
| putamen | 24 | 21 | 6 |  | 3.4 |
| putamen | 21 | 15 | 0 |  | 3.4 |
| precentral gyrus | 48 | 0 | 48 | 17 | 3.5 |
| precentral gyrus | 51 | 9 | 24 | 13 | 3.4 |
|  |  |  |  |  |  |
| seed: left vpIFG * |  |  |  |  |  |
| inferior parietal gyrus | 57 | -57 | 33 | 32 | 3.74 |
| precentral gyrus | 48 | 3 | 48 | 14 | 3.68 |
| cerebellum | 30 | -54 | -27 | 32 | 3.6 |
| cerebellum | 21 | -51 | -27 |  | 3.43 |
| putamen | -21 | 0 | 6 | 19 | 3.53 |
| putamen | -24 | 6 | -3 |  |  |

All activation peaks were thresholded at p<0.05, FDR corrected, except

* PPI coordinates of right and left vpIFG were reported at a lower threshold, p<0.001, uncorrected; cluster size >= 9 voxels.
