# Supplementary material for: Rule-Guided Executive Control of Response Inhibition: Functional Topography of the Inferior Frontal Cortex
Source: PLoS One. 2011 Jun 6;6(6):e20840. doi: 10.1371/journal.pone.0020840 (PMC3108978; doi:10.1371/journal.pone.0020840)
Supplement: Table S3 — List of PPI seed coordinates for individuals. (DOC) [file pone.0020840.s003.doc]

Supplementary Table 3 List of PPI seed coordinates for individuals

| subject | x | y | z |
| --- | --- | --- | --- |
| left aIFG |  |  |  |
| 1 | -45 | 45 | 9 |
| 2 | - | - | - |
| 3 | - | - | - |
| 4 | -48 | 45 | 9 |
| 5 | -36 | 48 | 9 |
| 6 | - | - | - |
| 7 | -39 | 45 | 12 |
| 8 | -48 | 42 | 9 |
| 9 | -48 | 36 | 0 |
| 10 | -45 | 45 | 12 |
| 11 | -39 | 42 | 12 |
| 12 | -48 | 45 | 3 |
| 13 | -33 | 48 | 9 |
| 14 | -33 | 51 | 9 |
| 15 | -48 | 45 | 12 |
| 16 | -48 | 51 | 3 |
| 17 | -27 | 57 | 12 |
| 18 | - | - | - |
| 19 | - | - | - |
| 20 | -45 | 48 | 0 |
| 21 | -39 | 36 | -3 |
| 22 | - | - | - |
| 23 | -42 | 48 | 9 |
|  |  |  |  |
| right dpIFG |  |  |  |
| 1 | 39 | 15 | 24 |
| 2 | 45 | 15 | 33 |
| 3 | 51 | 12 | 30 |
| 4 | 54 | 21 | 27 |
| 5 | 54 | 12 | 30 |
| 6 | 51 | 12 | 36 |
| 7 | 57 | 12 | 30 |
| 8 | 51 | 15 | 33 |
| 9 | 57 | 21 | 24 |
| 10 | 48 | 12 | 33 |
| 11 | 54 | 12 | 30 |
| 12 | 57 | 21 | 30 |
| 13 | 48 | 12 | 33 |
| 14 | 54 | 21 | 27 |
| 15 | 51 | 12 | 33 |
| 16 | 48 | 6 | 27 |
| 17 | - | - | - |
| 18 | 45 | 21 | 33 |
| 19 | 48 | 6 | 33 |
| 20 | 45 | 18 | 15 |
| 21 | 54 | 12 | 24 |
| 22 | 54 | 9 | 21 |
| 23 | 54 | 12 | 42 |
|  |  |  |  |
| right vpIFG |  |  |  |
| 1 | 51 | 18 | 3 |
| 2 | 45 | 21 | 0 |
| 3 | 54 | 24 | -6 |
| 4 | 45 | 21 | 0 |
| 5 | 51 | 21 | 0 |
| 6 | 48 | 18 | -3 |
| 7 | 51 | 30 | -6 |
| 8 | 45 | 18 | 3 |
| 9 | 45 | 21 | 0 |
| 10 | 48 | 24 | -6 |
| 11 | 48 | 18 | -3 |
| 12 | 51 | 21 | 6 |
| 13 | 51 | 15 | -3 |
| 14 | 48 | 18 | -3 |
| 15 | 42 | 27 | 6 |
| 16 | 45 | 21 | 6 |
| 17 | 45 | 18 | 3 |
| 18 | 48 | 18 | -3 |
| 19 | 45 | 21 | 0 |
| 20 | - | - | - |
| 21 | 45 | 21 | 0 |
| 22 | 45 | 21 | 0 |
| 23 | 45 | 21 | 0 |
|  |  |  |  |
| left vpIFG |  |  |  |
| 1 | -54 | 24 | 0 |
| 2 | -54 | 12 | 0 |
| 3 | -54 | 21 | -3 |
| 4 | -42 | 18 | -6 |
| 5 | - | - | - |
| 6 | -39 | 21 | -3 |
| 7 | -45 | 12 | 0 |
| 8 | -45 | 12 | 3 |
| 9 | -42 | 15 | -6 |
| 10 | -45 | 18 | 3 |
| 11 | - | - | - |
| 12 | -54 | 18 | 3 |
| 13 | - | - | - |
| 14 | -39 | 18 | 6 |
| 15 | -48 | 18 | 3 |
| 16 | -54 | 9 | 6 |
| 17 | -45 | 18 | -3 |
| 18 | -48 | 12 | -3 |
| 19 | -39 | 18 | -6 |
| 20 | - | - | - |
| 21 | - | - | - |
| 22 | - | - | - |
| 23 | - | - | - |

Note: The seeds were individually defined and centered at the peak activation of the IFG subregions. Some individual seeds were missing because no suprathreshold activation was found in the region at p<0.05, uncorrected.
