# Supplementary material for: Rule-Guided Executive Control of Response Inhibition: Functional Topography of the Inferior Frontal Cortex
Source: PLoS One. 2011 Jun 6;6(6):e20840. doi: 10.1371/journal.pone.0020840 (PMC3108978; doi:10.1371/journal.pone.0020840)
Supplement: Table S4 — List of clusters and coordinates from the contrast of SST-NST response blocks (using block GLM). (DOC) [file pone.0020840.s004.doc]

Supplementary Table 4 List of clusters and coordinates from the contrast of SST-NST response blocks (using block GLM)

| regions | X | Y | Z | cluster size | Z |
| --- | --- | --- | --- | --- | --- |
| cerebellum | -6 | -81 | -30 | 1468 | 5.96 |
| cerebellum | -30 | -69 | -33 |  | 5.82 |
| cerebellum | -39 | -63 | -36 |  | 5.47 |
| inferior parietal lobule | 48 | -42 | 42 | 1263 | 5.49 |
| inferior parietal lobule | 42 | -60 | 51 |  | 5.33 |
| inferior parietal lobule | 33 | -54 | 36 |  | 5.22 |
| middle frontal gyrus | 42 | 33 | 30 | 4775 | 5.45 |
| dorsal-posterior inferior frontal gyrus / inferior frontal junction | -45 | 0 | 27 |  | 5.32 |
| presupplementary motor area / cingulated gyrus | 6 | 15 | 48 |  | 5.26 |
| superior parietal lobule | -18 | -69 | 48 | 1089 | 5.12 |
| inferior parietal lobule | -36 | -45 | 36 |  | 5.01 |
| inferior parietal lobule | -27 | -51 | 36 |  | 4.66 |
| inferior frontal gyrus | -30 | 24 | 0 | 194 | 4.07 |
| middle frontal gyrus | -36 | 48 | 21 | 144 | 3.83 |
| anterior inferior frontal gyrus | -39 | 54 | 12 |  | 3.77 |
| middle frontal gyrus | -33 | 39 | 21 |  | 3.65 |
| Posterior cingulate gyrus | 3 | -27 | 24 | 40 | 3.44 |
| lingual gyrus | 18 | -90 | -3 | 69 | 3.19 |
| lingual gyrus | 18 | -72 | 6 |  | 2.77 |
| middle temporal gyrus | 57 | -27 | -15 | 11 | 3.15 |
| lingual gyrus | -9 | -75 | 6 | 18 | 2.77 |
| middle temporal gyrus | 54 | -42 | 0 | 14 | 2.73 |
|  |  |  |  |  |  |
| Inferior frontal gyrus sub-clusters |  |  |  |  |  |
| dorsal-posterior inferior frontal gyrus | -45 | 0 | 27 |  | 5.32 |
| ventral-posterior inferior frontal gyrus | 48 | 18 | 0 |  | 5.03 |
| dorsal-posterior inferior frontal gyrus | 51 | 9 | 33 |  | 4.04 |
| ventral-posterior inferior frontal gyrus | -39 | 18 | -6 |  | 3.79 |
| anterior inferior frontal gyrus | -39 | 54 | 12 |  | 3.77 |

These IFG clusters from the group contrast map (p<0.05, FDR corrected) were used to guide the identification of seeds in individual subject for PPI analysis.
